# Investigating the Effects of Dehydrated Human Amnion-Chorion Membrane on Periodontal Healing

**DOI:** 10.3390/biom12060857

**Published:** 2022-06-20

**Authors:** Kentaro Imamura, Yusuke Hamada, Wataru Yoshida, Tasuku Murakami, Saki Nakane-Koyachi, Kouki Yoshikawa, Atsushi Saito

**Affiliations:** 1 Department of Periodontology, Tokyo Dental College, Chiyoda-ku, Tokyo 101-0061, Japan; yoshidawataru@tdc.ac.jp (W.Y.); murakamitasuku@tdc.ac.jp (T.M.); nakanesaki@tdc.ac.jp (S.N.-K.); yoshikawakouki@tdc.ac.jp (K.Y.); atsaito@tdc.ac.jp (A.S.); 2 Oral Health Science Center, Tokyo Dental College, Chiyoda-ku, Tokyo 101-0061, Japan; 3 Department of Periodontology, School of Dentistry, Indiana University, Indianapolis, IN 46202, USA; yuhamada@iupui.edu

**Keywords:** amnion-chorion membrane, placenta, periodontitis, periodontal treatment, cell proliferation, angiogenesis, growth factors

## Abstract

Each growth factor (GF) has different effects and targets, and plays a critical role in periodontal healing. Dehydrated human amnion-chorion membrane (dHACM) contains various GFs and has been used to enhance wound healing. The purpose of this study was to evaluate the effects of dHACM on periodontal healing, using in vitro and in vivo experimental approaches. Standardized periodontal defects were created in rats. The defects were randomly divided into three groups: Unfilled, filled with hydroxypropyl cellulose (HPC), and dHACM+HPC. At 2 and 4 weeks postoperatively, periodontal healing was analyzed by microcomputed tomography (micro-CT), and histological and immunohistochemical analyses. In vitro, periodontal ligament-derived cells (PDLCs) isolated from rat incisors were incubated with dHACM extract. Cell proliferation and migration were evaluated by WST-1 and wound healing assay. In vivo, micro-CT examination at 2 weeks revealed enhanced formation of new bone in the dHACM+HPC group. At 4 weeks, the proportions of vascular endothelial growth factor (VEGF)-positive cells and α-smooth muscle actin (α-SMA)-positive blood vessels in the dHACM+HPC group were significantly greater than those in the Unfilled group. In vitro, dHACM extracts at 100 µg/mL significantly increased cell proliferation and migration compared with control. These findings suggest that GFs contained in dHACM promote proliferation and migration of PDLCs and angiogenesis, which lead to enhanced periodontal healing.

## 1. Introduction

The purpose of periodontal regenerative therapy is to restore tooth supporting structures that have been lost as a result of periodontitis [1]. A successful outcome of periodontal regeneration requires the following essential factors: appropriate cells, signals, scaffolds, blood supply, mechanical loading, and pathogen control [2]. To date, the use of biologics such as enamel matrix derivative (EMD), recombinant human fibroblast growth factor (rhFGF)-2 produces clinically favorable periodontal healing [3].

The human placenta consists of two fetal sheaths: the outer chorion and the inner amnion membranes [4]. Early use of fresh amniotic membrane containing both amnion and chorion has proved beneficial in treating ulcers, burns and dermal injuries [5]. In addition, human amnion is known to be non-immunogenic, to reduce inflammation, pain and scarring and provide a matrix for cell colonization as well as a natural biological barrier [6].

Human amnion-chorion membrane (HACM) not only works as a protective wound barrier, but also provides a biological matrix that supports cell proliferation and tissue ingrowth [7,8]. HACM contains many types of growth factor (GF)s, such as fibroblast GF (FGF), epidermal GF (EGF), and transforming GF-α (TGFα) [9]. These GFs are known to play critical roles in the physiological processes leading to wound healing and tissue regeneration [10]. Besides, HACM has a rich inheritance of collagen types I, IV, V, and VI, proteoglycans, laminin, and fibronectin and the potential to promote revascularization and tissue healing [11].

Dehydrated human amnion-chorion membrane (dHACM) contains angiogenic GFs retaining biological activity and promotes amplification of angiogenic cues by inducing endothelial cell proliferation and migration and by enhanced production of endogenous angiogenic GFs [12]. There have been several reports focused on the use of dHACM, which is used in this experiment for procedures such as extraction socket preservation [13] and guided bone regeneration [14]. Additionally, some periodontal case reports showed that the dHACM could promote periodontal healing in intrabony defects [15,16]. However, information is still limited on the effects of dHACM on periodontal healing and its mechanisms of action. We hypothesized that the GFs in dHACM enhance periodontal healing via promotion of cell proliferation and differentiation. This study aims to evaluate the effects of dHACM on periodontal healing, using in vivo and in vitro experimental approaches.

## 2. Materials and Methods

### 2.1. dHACM

A minimally manipulated dHACM processed from placental tissues (BioXclude^®^, Snoasis, Denver, CO, USA) was used for this study.

### 2.2. Animals

Male Wistar rats (in vitro experiments: 4 weeks old, in vivo experiments: 10 weeks old) were obtained from Sankyo Labo Service (Tokyo, Japan). Rats were maintained under standard laboratory conditions. The study adhered to the ARRIVE guidelines (https://www.nc3rs.org.uk/arrive-guidelines accessed on 17 May 2022) and the treatment of experimental animals at Tokyo Dental College (approval no. 212205).

### 2.3. Surgical Procedures

These defects were allocated to one of the following 3 subgroups: (1) dHACM and hydroxypropyl cellulose (HPC) (*n* = 10), (2) HPC only (*n* = 10), (3) Unfilled (*n* = 10). Randomization was performed using computer-generated allocation, by one of the coauthors who was not involved in the assessment. Local anesthesia was used and full-thickness flaps were raised. Then, bilateral standardized periodontal defects (2 mm × 2 mm × 1.7 mm, width × length × depth) [17] were created mesially of the maxillary first molars (M1) (Figure S1; Supplementary File). The root of M1 was denuded of its PDL, cementum and superficial dentin. The defects received dHACM and HPC, HPC only, or left unfilled. Unlike the barrier membrane used in the conventional guided tissue regeneration (GTR) method, which is placed over the defect area, dHACM was placed onto the root surface below cemento-enamel junction so as not to deviate from the created bone defect. The flaps were closed using resorbable sutures (Vicryl^®^ 5-0; Ethicon Products, Tokyo, Japan). Acetaminophen was used for postoperative pain control.

### 2.4. Microcomputed Tomography Analysis

At 2 and 4 weeks, the animals were anesthetized and cardiovascular perfusion was performed with 4% paraformaldehyde. Maxillae were retrieved, and the healing in the surgical defect was examined using image data obtained by microcomputed tomography (micro-CT) system (R-mCT; Rigaku, Tokyo, Japan). The exposure conditions were 90 kV and 150 µA. The magnification was set at ×10, and a slice width was 16 µm. The information from all the slices was saved at 484 × 481 pixels as described previously [18]. Bone volume fractions and trabecular number, thickness, and separation of the newly formed bone within the defect were analyzed using 3-D structural analysis software (TRI/3D-BON; Ratoc System Engineering, Tokyo, Japan) by the method described by Bizenjima et al. [19].

### 2.5. Histological Processing

Subsequently, the maxillae were split into two parts through the palatal median line. After fixation in buffered 4% paraformaldehyde for 24 h, samples were decalcified in 10% EDTA at 4 °C for 3 weeks and then embedded in paraffin. Mesio-distal sections (thickness 5 µm) were cut with a microtome (Hyrax M25, Carl Zeiss, Jena, Germany). From each specimen, five to ten sections representing the central portion of the root in the defect were stained with hematoxylin-eosin or Azan–Mallory staining for histological and histomorphometric analyses.

### 2.6. Histomorphometric Analysis

The following measurements were made under a light microscope (UPM Axiophot 2; Carl Zeiss Japan, Tokyo, Japan) with histomorphometric software (Axio Vision 4.7; Carl Zeiss, Tokyo, Japan), as described previously [19]: (1) root length, (2) length of the junctional epithelium, measured as the distance between the most coronal and most apical aspects of the epithelium along the root, and (3) the distance between the cemento-enamel junction and the bottom of the defect.

On the root planed surface of M1, the angulation of PDL-like fibers was analyzed by Image J software (http://rsb.info.nih.gov/ij accessed on 1 February 2022), as described by Park et al. [20].

### 2.7. Immunohistochemistry

Paraffin sections were deparaffinized and incubated in 3% hydrogen peroxide with methanol for 30 min at room temperature. The sections were treated with a microwave oven for 3 min. After washing with PBS, the sections were incubated with 3% bovine serum albumin for 30 min to block nonspecific binding.

For the assessment of angiogenesis, immunohistochemical detection of vascular endothelial growth factor (VEGF) and α-smooth muscle actin (SMA, a marker for vessel wall or pericytes) were performed. VEGF expression was examined using anti-VEGF monoclonal antibodies (1:50; Abcam ab1316, Tokyo). Anti-α-SMA monoclonal antibodies (dil. 1:100; Abcam ab7817) were used. Immunostaining of cells in sub-epithelial connective tissue was assessed. To confirm specificity of staining a non-immune mouse IgG (Abcam) control was used at the same concentration. Cells positive for VEGF was counted under a light microscope at × 200 magnification and a field of connective tissue (350 µm × 470 µm area) was randomly sampled in each section using the image analyzer, and their number was expressed as a proportion of positive cells [19,21]. VEGF positive cells were counted in the area adjacent to the M1 mesial root above the newly formed bone. Cell counts were obtained by one examiner and confirmed by a second independent examiner with similar results. The percentage of α-SMA positive blood vessels to that of total blood vessels was calculated as described previously [19,22].

### 2.8. Preparation of dHACM Extract

dHACM (cut to 2 × 2 mm; 200 µg) and 150 µL of phosphate buffered saline (PBS; pH 7.0) were placed in a mortar cooled with liquid nitrogen. The mixture was ground by pestle with a gloved hand and firmly pressed on the sample while twisting. After grinding, the debris contained in the mixture was removed by centrifugation to produce dHACM extract.

The concentration of VEGF and fibroblast growth factor (FGF)-2 in dHACM extract was measured by enzyme-linked immunosorbent assay (ELISA) for human VEGF (Funakoshi, Tokyo, Japan) and FGF-2 (Funakoshi). Experiments were performed according to the manufacturer’s protocol. The lower limitation of detection was 9 pg/mL for VEGF and 3 pg/mL for FGF-2.

### 2.9. Isolation of Rat Periodontal Ligamint-Derived Cells (rPDLCs) and Culturing Method

For evaluation of the effect of dHACM extract on cell viability/proliferation and cell migration, rat periodontal ligament (PDL)-derived cells (rPDLCs) were obtained from incisors of 4-week-old male Wistar rats by the method of Inoue [23]. Briefly, the crowns of the teeth were resected and the roots placed in medium containing the antibiotic solution described above. The roots were bisected axially by surgical blade, after which the pulp tissue was removed by mechanical means. The root fragments and their adhering PDL were placed in α-minimal essential medium (αMEM, Gibco, Invitrogen, Carlsbad, CA, USA) for one hour. Cells were maintained in αMEM, supplemented with heat-inactivated 10% fetal bovine serum and antimicrobials at 37 °C in 5% CO_2_ in humidified air.

### 2.10. Cell Viability/Proliferation and Wound Healing Assays

Cell viability/proliferation of dHACM extract was evaluated by WST-1 assay. rPDLCs (10^5^ cells/well) in 200 µL of culure media were seeded in 96-well plates and incubated for 3 days. After incubation, cells were incubated with some concentration (100 to 500 µg/mL) of dHACM extract for 48 h. In these experiments, 500 µg/mL of dHACM (250 µg of dHACM and 500 µL of culture media) was prepared, diluted and used.

The effect of dHACM extract on cell migration was evaluated by wound healing assay. rPDL cells were incubated in 24-well plates, after 72 h, three artificial wounds per six well plate were made using a pipette tip, as described by Imamura et al. [24]. The sizes of the wound were nearly constant (approximately 500 µm in width) at the beginning of cell migration. The cells were washed quickly to remove the floating detached cells. The wound area was monitored for up to 24 h using a fluorescence microscope (BZ-X710; Keyence, Kyoto, Japan). The cells were maintained in a tissue culture incubator between image acquisitions. The captured images were tracked by using an image processing program (Image J; National Institutes of Health, Bethesda, MD, USA) (http://rsb.info.nih.gov/ij accessed on 1 February 2022) that measured the area of the in vitro wound. Six consecutive microscopic fields were analyzed and averaged. The wound closure percentage was calculated by dividing the wound area (0–24 h) by the original area.

### 2.11. Statistical Analysis

The sample size was determined by a power analysis based on 90% power with a 0.05 two-sided significance level, given a difference in bone volume between groups of 10.5% (as measured by micro-CT) and a standard deviation of 7% [19,20], using a software package (StatMate 2 for Windows, GraphPad Software, La Jolla, CA, USA). According to the determination, a sample size of 10 (defect site) in each group was needed.

Comparisons between groups were performed by analysis of variance (ANOVA) with Tukey post test or Kruskal-Wallis test with Dunn’s post test. A *p*-value less than 0.05 was considered statistically significant. A software package (InStat 3.10, GraphPad Software, San Diego, CA, USA) was used. A *p*-value less than 0.05 was considered statistically significant.

## 3. Results

### 3.1. Micro-CT Analysis of Defect Healing

**Figure 1:**
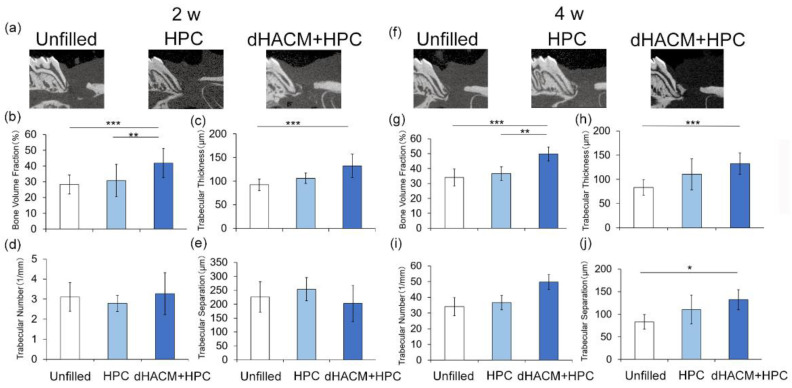
Two-dimensional images in micro-CT and quantitative analysis by 3-D structural analysis software (TRI/3D-BON). (**a**,**f**) A sagittal slice images from micro-CT at 2 and 4 weeks. Enhanced newly formed bone can be observed in the dHACM+HPC group at 2 and 4 weeks. (**b**–**e**,**g**–**j**) Quantitative analysis of micro-CT images by a 3-D structural analysis software (TRI/3D-BON). White bar, Unfilled group; Light blue bar, HPC group; Blue bar, dHACM+HPC group. bone volume fraction (**b**,**g**), trabecular number (**c**,**h**), trabecular separation (**d**,**i**) and thickness (**e**,**j**) were compared between groups. Data shown as mean ± SD (*n* = 10). * *p* < 0.05, ** *p* < 0.01, *** *p* < 0.001 by Kruskal-Wallis test with Dunn’s post hoc test.

Morphological healing was observed, and alveolar bone formation was measured by micro-CT. Sagittal images were shown in Figure 1a,f. At 2 and 4 weeks, newly formed bone was observed in the dHACM+HPC group (Figure 1f). Quantitative analysis at 2 weeks (Figure 1b–e) and 4 weeks (Figure 1g–j) showed that there were increases in bone volume fraction and trabecular thickness and decreases in trabecular separation for all groups.

At 2 weeks, bone volume fractions were significantly greater in the dHACM+HPC group compared with that in the Unfilled (*p* < 0.001) and HPC group (*p* < 0.01) (Figure 1b). At 4 weeks, bone volume fractions were significantly greater in the dHACM+HPC group compared with that in the Unfilled and HPC group similar to the results of 2 weeks (Figure 1g). The trabecular thickness in the dHACM+HPC group at 2 weeks was significantly greater than that in the HPC group (*p* < 0.05) (Figure 1c). At 4 weeks, trabecular thickness in dHACM+HPC group was significantly greater than in the Unfilled group (*p* < 0.001) (Figure 1h). There were no significant changes in trabecular number at 2 and 4 weeks (Figure 1d,i). At 4 weeks, the trabecular separation was significantly smaller in the dHACM+HPC group compared with that in the Unfilled group (*p* < 0.05) (Figure 1j). It was suggested that dHACM promoted bone healing.

### 3.2. Histological Observations

**Figure 2:**
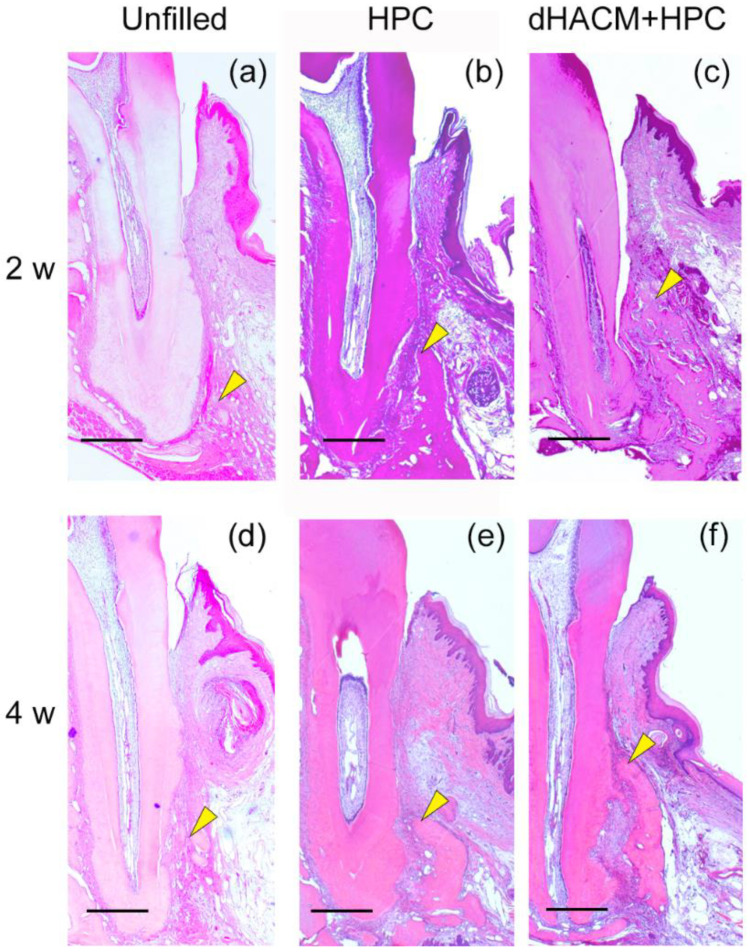
Histopathological overview (H-E staining). At 2 weeks, spaces in the defect are filled with newly formed connective tissue. (**a**,**b**) New bone formation is not observed in the Unfilled and HPC group. (**c**) In the dHACM+HPC group, newly formed bone can be observed from the tooth side of the intrabony defect. (**d**) At 4 weeks, new bone formation appeared minimal in the Unfilled group. (**e**) Newly formed bone was observed near the root side of the defect in the HPC group. (**f**) The extent of newly formed bone in the defect area in the dHACM + HPC group appeared to be greater than other groups. Yellow arrowheads indicate the coronal extent of newly formed bone. Original magnification ×25, bar = 500 µm.

Histological overviews are shown in Figure 2. he defect spaces were filled with the newly formed connective tissue in all groups (Figure 2a–f). There were no obvious signs of inflammation in the defect. In the Unfilled group, newly formed bone was limited at 2 and 4 weeks (Figure 2a,d). Bone formation appeared to be greater in the dHACM+HPC group compared with other group at 2 and 4 weeks.

### 3.3. Histomorphometric Analysis

**Figure 3:**
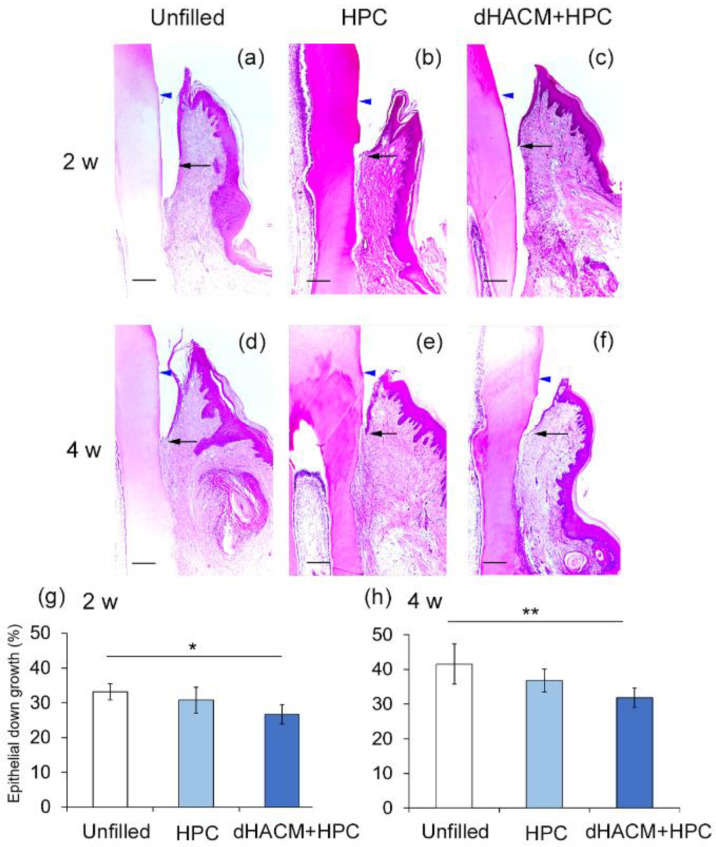
Histological assessment of epithelial down-growth. (**a**–**f**) The apical extent of epithelial down-growth (indicated by arrows) was compared at 2 and 4 weeks. Blue arrowheads indicate cemento-enamel junction (CEJ). (**a**,**d**) At 2 weeks, epithelial down-growth along the root surface in the dHACM+HPC group appears to be less than that in the Unfilled group (original magnification ×50; bar = 200 μm). (**g**,**h**) Data shown as mean ± SD (*n* = 6) of (the length between the most coronal and most apical aspects of the junctional epithelium on the root surface)/(the distance between CEJ and the bottom of the defect) (%) at 2 and 4 weeks. * *p* < 0.05, ** *p* < 0.01, by Kruskal–Wallis test with Dunn’s post hoc test.

Epithelial down-growth in the dHACM+HPC and HPC group was limited compared with that in the Unfilled group at 2 and 4 weeks, (Figure 3a–f). The relative length of the junctional epithelium was significantly shorter in dHACM+HPC group than in the Unfilled group at 2 and 4 weeks (Figure 3g,h).

**Figure 4:**
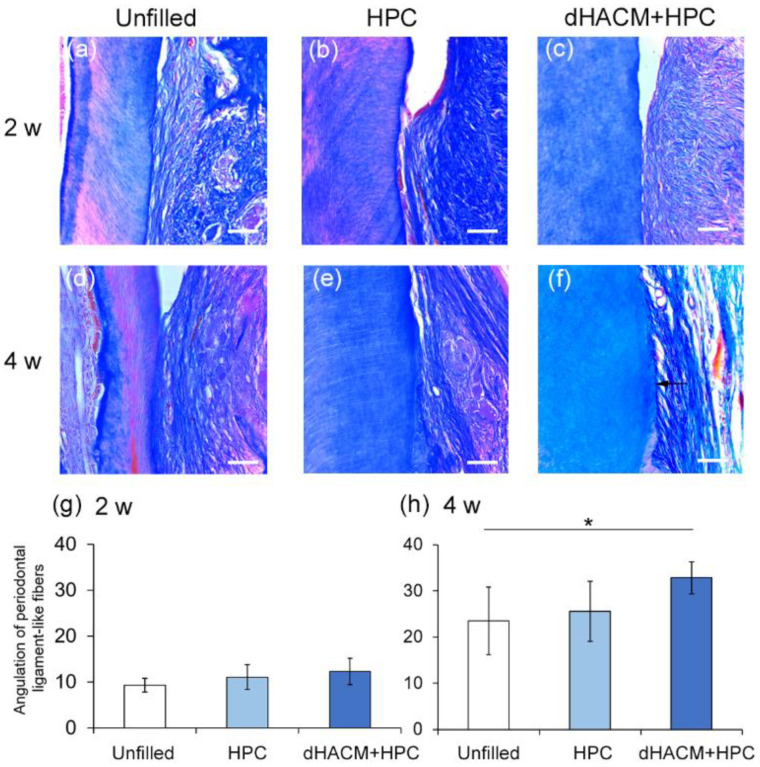
Healing of periodontal ligament (PDL). (**a**–**f**) Representative photomicrographs of the root area near the bottom of defect at 2 and 4 weeks. (**a**–**c**) At 2 weeks (upper panel), collagen bundles run near parallel to the surfaces in all groups. (**f**) At 4 weeks, PDL-like collagen bundles are well-aligned and obliquely inserted onto the root surface similar to native PDL with signs of thin-layer of cementogenesis (indicated by an arrow). (Azan–Mallory’s stain, original magnification ×200; bar = 50 μm). (**g**,**h**) The angulation of the fibers at the bottom area of instrumentation on M1 root was observed under ×200 and analyzed by Image J software at 2 and 4 weeks. Data shown as mean ± SD (*n* = 6) in degree. * *p* < 0.05 by ANOVA with Tukey post-test.

The healing of PDL was assessed by Azan–Mallory staining. Representative micrographs of Azan–Mallory staining of the areas near the defect bottom are shown in Figure 4a–f. PDL-like bundles in all groups were almost parallel to the root surfaces at 2 weeks, and cementum formation was not observed (Figure 4a–c). At 4 weeks, the PDL-like bundles ran obliquely in the dHACM+HPC group (Figure 4f). In the other groups, the ligaments ran near parallel to the root (Figure 4d,e). A thin layer of newly formed cementum-like structure was observed only in the dHACM + HPC group at 4 weeks. No significant difference in the mean angulation of the fiber bundles was observed at 2 weeks. The mean angulation in the dHACM + HPC group was significantly greater than that in Unfilled groups at 4 weeks. The application of dHACM promoted healing of PDL and, possibly, cementum.

### 3.4. Immunohistochemical Analysis

**Figure 5:**
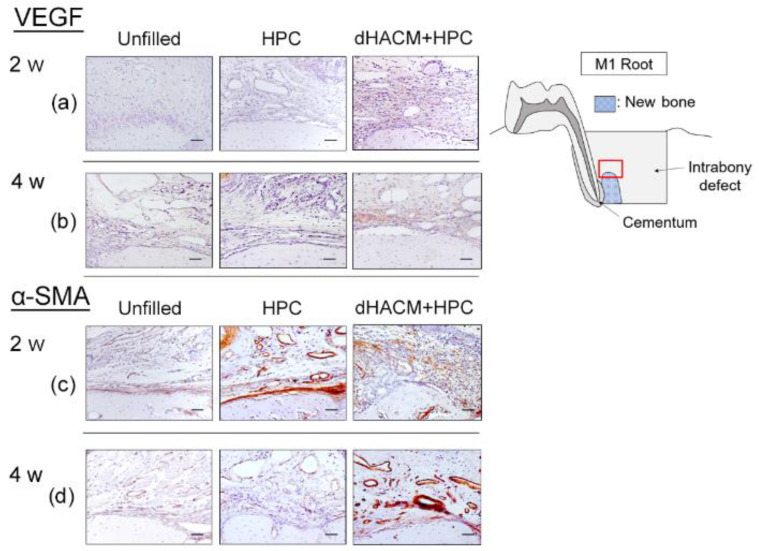
Representative photomicrographs of immunohistochemical staining for VEGF and α-SMA. VEGF-stained images at 2 weeks (**a**) and 4 weeks (**b**). A brown coloration indicates VEGF-positive reaction. The prevalence of VEGF-positive cells in the dHACM+HPC group appears to be greater than that in the Unfilled group. α-SMA-stained images at 2 weeks (**c**) and 4 weeks (**d**). The prevalence of α-SMA-positive blood vessels appears to be higher in the dHACM+HPC group compared with the Unfilled group (VEGF and α-SMA counterstaining with Mayer’s haematoxylin stain, original magnification ×200; bar = 50 μm).

The effect of application of dHACM for periodontal defect on angiogenesis was evaluated by assessing the immunohistochemical staining for VEGF and α-SMA. VEGF-positive cells were often observed close to blood vessels in the connective tissue and around newly formed bone (Figure 5). At 2 weeks, a significantly greater proportion of VEGF-positive cells was observed in the dHACM+HPC group compared with the Unfilled group (Table 1). At 4 weeks, the proportion of VEGF-positive cells in the dHACM+HPC group was significantly greater than that in the HPC and Unfilled group (*p* < 0.05). At 4 weeks, the proportion of VEGF-positive cells in the dHACM+HPC group was significantly greater than that in the Unfilled group.

In α-SMA immunohistochemical analysis, the prevalence of α-SMA-positive cells in dHACM+HPC group appeared to be higher compared with other groups at 4 weeks (Figure 5). Quantitative analysis showed no significant difference in the proportions of α-SMA-positive blood vessels between groups at 2 weeks (Table 1). However, at 4 weeks, the proportion of α-SMA-positive blood vessels was significantly greater in the dHACM+HPC group compared with other groups (*p* < 0.05).

### 3.5. The Concentration of VEGF and FGF-2 in the dHACM Extract

The concentrations of GFs in dHACM extract were measured by ELISA. The dHACM extract contained 50 pg/mL of VEGF and 23 pg/mL of FGF-2.

### 3.6. The Effect of dHACM Extract on Cell Proliferation

**Figure 6:**
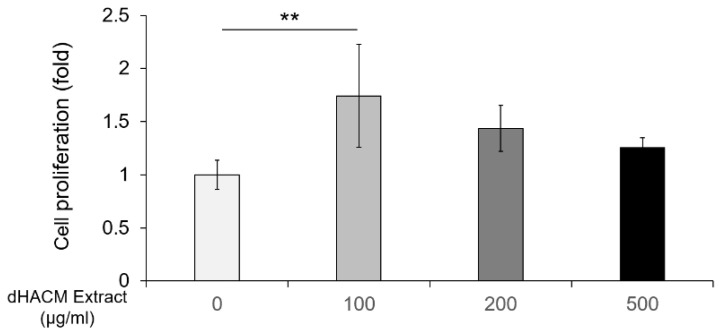
The effect of dHACM on cell proliferation. After incubation with/without dHACM extract for 48 h, the proliferation of rPDLCs was assessed by WST-1. Values are shown as means ± standard deviations (*n* = 9). ** *p* < 0.01, compared to the untreated control, ANOVA with Tukey post-test.

The effect of dHACM extract on viability/proliferation of rPDLCs, isolated from rat incisors, was evaluated by WST-1 assay. The level of cell proliferation was significantly greater (1.7-fold, *p* < 0.01) in 100 µg/mL of dHACM extract compared with the control (Figure 6). There was no significant difference in cell proliferation between nontreated control group and some concentration of 200 and 500 µg/mL of dHACM extract. The concentration range (100–500 µg/mL) of dHACM extract showed no significant cytotoxic effects. However, it was confirmed that higher concentrations (1000 µg/mL) of dHACM extract had cytotoxicity.

### 3.7. The Effect of dHACM Extract on Cell Migration

**Figure 7:**
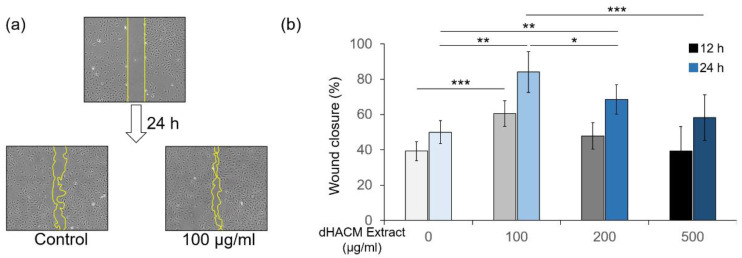
The effect of dHACM on cell migration. rPDLC monolayers were cultured to confluence, and various concentrations of dHACM extract were added and further incubated. Then multiple artificial wounds were made, and the wound area was monitored up to 12 and 24 h. Representative microphotographs of the wound closure after incubation with/without dHACM extract for 24 h (**a**). The rate of wound closure of rPDLCs treated with/without dHACM extract (**b**). This was obtained using the following formula: (initial wound area)—(wound area after an identified culture period)/(initial wound area) × 100. Values are shown as means ± standard deviations (*n* = 9). * *p* < 0.05, ** *p* < 0.01, and *** *p* < 0.001, compared to the untreated control, ANOVA with Tukey post-test.

In the wound healing assay, dHACM extract at 100–200 µg/mL promoted wound closure of rPDLC in 24 h (Figure 7). The 500 µg/mL of dHACM extract showed no significant the effect on cell migration.

## 4. Discussion

To the best our knowledge, this is the first study to demonstrate in vivo and in vitro the effects of dHACM on periodontal healing. The in vivo results showed that the dHACM promoted the healing of periodontal defects in rats. In vitro, the dHACM extract promoted proliferation and migration of rPDLCs, possibly mediated by GFs such as VEGF and FGF-2 contained in dHACM.

In micro-CT analysis, the values for bone volume fraction in the dHACM+HPC group were significantly greater than in the Unfilled and HPC alone groups at 2 and 4 weeks. Also, in the histological evaluation, the dHACM+HPC group promoted newly formed bone and inhibited epithelial down-growth. In addition, the angulation of the PDL-like collagen bundles in dHACM+HPC group was similar to that of the native PDL-fibers [21]. PDL-like bundles ran oblique to the root surface, suggesting functional reorganization of the PDL in relation to connective tissue attachment. In previous studies, the positive effects of the dHACM on periodontal tissue healing have been clinically reported [16,25]. Therefore, it is considered that two factors, the activation of PDLCs and the suppression of epithelial down-growth, may have been involved in the promotion of periodontal healing by the dHACM. Guided tissue regeneration (GTR) promotes new connective tissue attachment and cementum formation [26]. Gingival epithelial cells were prevented from migrating into a wound by means of a membrane barrier, and at the same time, PDLCs were increased for coronal migration. In the current experimental model, it was possible that an event similar to the GTR occurred due to the dHACM placed on the root surface. Conventional GTR is prone to clinical complications, including membrane exposure and gingival recession. In the present study, the dHACM is placed onto the root surface in the defect, which is less technically sensitive and less likely to cause clinical complications. However, there may be a disadvantage in creating an appropriate space, which is also needed in periodontal regeneration. This needs to be verified in future experiments.

Interestingly in our histological analysis, the dHACM remnant was not observed at 2 weeks postoperatively. The resorption time may vary depending on the biological environment, species, and membrane size, etc. It was reported that HACM contained many types of GFs [7,9]. In the present study, we confirmed the presence of FGF-2 and VEGF in the dHACM extract. FGF-2 promotes angiogenesis in combination with VEGF released from PDLCs [27]. At 2 weeks, greater proportion of VEGF-positive cells were observed in the dHACM+HPC group compared with Unfilled group. Furthermore, the prevalence of α-SMA-positive blood vessels in the dHACM+HPC group was significantly greater compared with other groups at 4 weeks. At the wound site, VEGF promotes the new vessel formations and increases vasopermeability [28,29]. Our previous studies reported that the application of FGF-2 to the experimental periodontal defects in rat increased VEGF positive cells at 2 weeks [20,30]. VEGF is used as a marker for angiogenesis. α-SMA is expressed in vessel wall or pericytes [31]. It can be used as a marker for pericyte differentiation. From these results of α-SMA immunostaining in the present study, we speculate that dHACM helped the newly forming blood vessels (capillaries) to mature. The angiogenesis helps the supply of nutrition, oxygen, and minerals essential for mineralization and osteogenesis. As a result, vascularization plays a key role in bone growth and healing. These findings collectively suggest that dHACM promoted angiogenesis, and this may also have contributed to enhanced periodontal healing.

One of the important functions of PDL is the formation of cementum [32]. In our histological evaluation, the newly formed cementum was not clearly observed. Because the cementum on rat tooth is extremely thin, it is difficult to observe newly formed cementum [20]. Azan–Mallory staining showed that the angulation of the fiber bundles in the dHACM+HPC group was significantly greater than that in Unfilled groups at 4 weeks. During the development of PDL, principal fiber aligns coronally from cementum to bone to form the oblique bundles [33]. PDL bundles may be inserted into newly cementum because the PDL-like bundles of dHACM group ran obliquely. Therefore, it was suggested that new cementum was formed near the PDL-like bundles in dHACM group.

## 5. Conclusions

This study demonstrated that the treatment with the dHACM yielded enhanced healing of surgically-created periodontal defects in rats. This may be attributed to the promotion of cell proliferation and migration, and angiogenesis induced by the GFs contained in dHACM. Although further research is necessary, our data provide important implications for the development of predictable periodontal regenerative therapy.

## Figures and Tables

**Table 1:** Quantitative analysis of VEGF and α-SMA immunohistochemical images.

Group		Unfilled	HPC	dHACM+HPC
VEGF	2 W	7.7 ± 1.6	10.5 ± 3.2	17.4 ± 3.1 ^a^
	4 W	10.3 ± 1.0	14.3 ± 2.5	17.9 ± 2.6 ^a^
α-SMA	2 W	9.7 ± 3.6	11.3 ± 3.0	12.0 ± 4.6
	4 W	11.8 ± 3.1	11.7 ± 4.5	20.7 ± 4.9 ^a,b^

Data shown as mean ± SD (*n* = 6) of VEGF-positive cells/total cells (%) and α-SMA-positive blood vessels/total cells (%). VEGF; vascular endothelial growth factor, α-SMA; α-smooth muscle actin. a; significantly different from the Unfilled group, b; significantly different from the HPC group, by Kruskal–Wallis test with Dunn’s post hoc test (*p* < 0.05).

## Data Availability

All data obtained in this research are described in the manuscript.

## Supplementary Materials

The following supporting information can be downloaded at: https://www.mdpi.com/article/10.3390/biom12060857/s1, Figure S1. Bilateral standardized periodontal defects (2 mm × 2 mm × 1.7 mm, width × length × depth) were created mesially of the maxillary first molars (M1).
