# Supplementary figures and images for: Investigating the Effects of Dehydrated Human Amnion-Chorion Membrane on Periodontal Healing
Source: Biomolecules. 2022 Jun 20;12(6):857. doi: 10.3390/biom12060857 (PMC9221211; doi:10.3390/biom12060857)

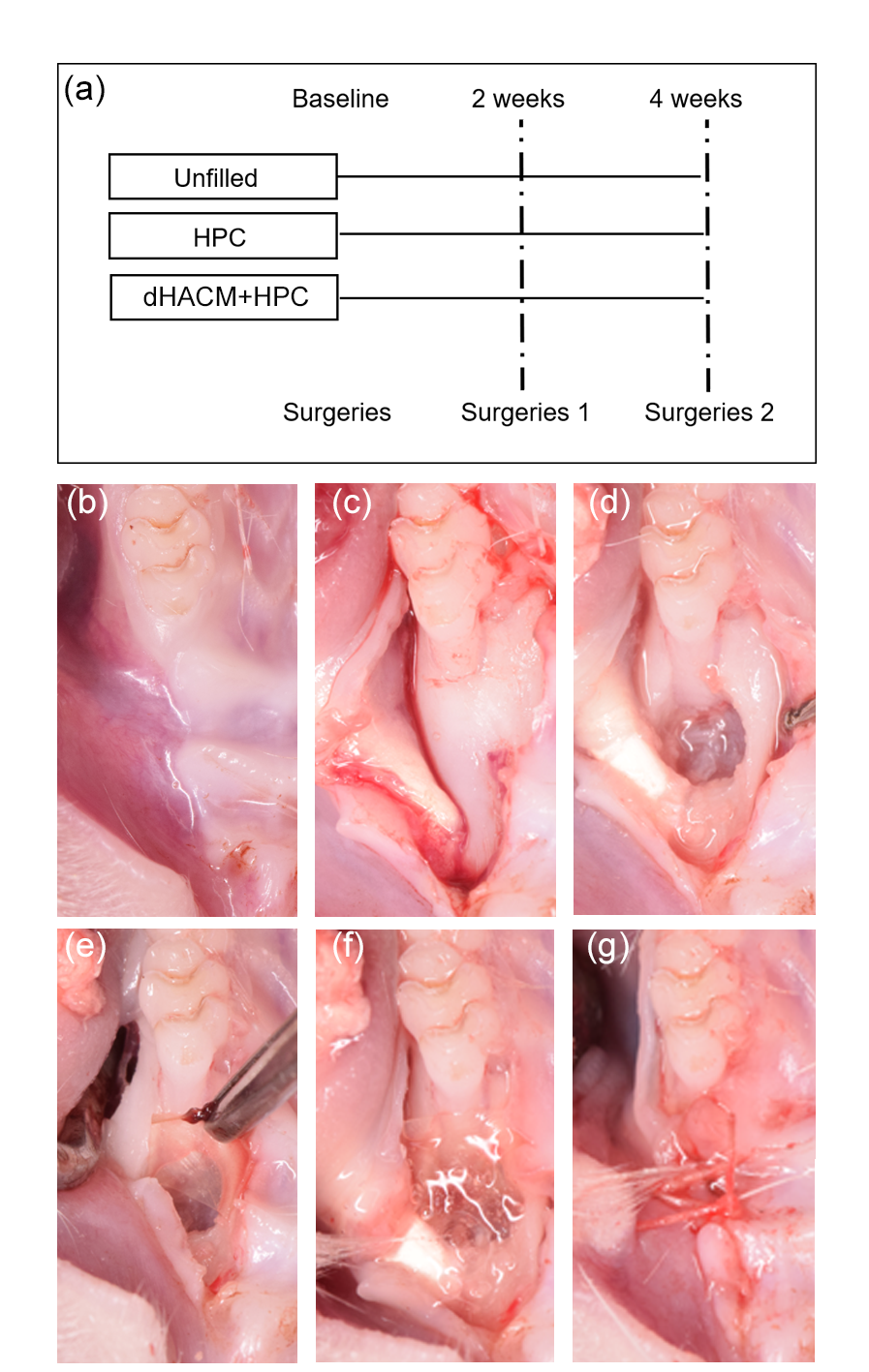

Supplement: Supplementary file 1 [file biomolecules-12-00857-s001.zip › Figure S1.tif]
